# Identification of a class of human cancer germline genes with transcriptional silencing refractory to the hypomethylating drug 5-aza-2′-deoxycytidine.

**DOI:** 10.18632/oncoscience.95

**Published:** 2014-11-10

**Authors:** Ahmed Almatrafi, Julia Feichtinger, Ellen G. Vernon, Natalia Gomez Escobar, Jane A. Wakeman, Lee D. Larcombe, Ramsay J. McFarlane

**Affiliations:** ^1^ North West Cancer Research Institute, School of Medical Sciences, Bangor University, Bangor, United Kingdom; ^2^ Current address: Department of Biology, Faculty of Science, University of Taibah, Medinah, Saudi Arabia; ^3^ Institute for Knowledge Discovery, Graz University of Technology, Graz, Austria; ^4^ Core Facility Bioinformatics, Austrian Centre of Industrial Biotechnology, Graz, Austria; ^5^ MRC Functional Genomics Unit, Department of Anatomy, Physiology and Genetics, University of Oxford, Oxford, United Kingdom

**Keywords:** cancer/testis antigen, germline gene, oncogenesis, hypomethylation, gene silencing

## Abstract

*Bona fide* germline genes have expression restricted to the germ cells of the gonads. Testis-specific germline development-associated genes can become activated in cancer cells and can potentially drive the oncogenic process and serve as therapeutic/biomarker targets; such germline genes are referred to as cancer/testis genes. Many cancer/testis genes are silenced via hypermethylation of CpG islands in their associated transcriptional control regions and become activated upon treatment with DNA hypomethylating agents; such hypomethylation-induced activation of cancer/testis genes provides a potential combination approach to augment immunotherapeutics. Thus, understanding cancer/testis gene regulation is of increasing clinical importance. Previously studied cancer/testis gene activation has focused on X chromosome encoded cancer/testis genes. Here we find that a sub-set of non-X encoded cancer/testis genes are silenced in non-germline cells via a mechanism that is refractory to epigenetic dysregulation, including treatment with the hypomethylating agent 5-aza-2′-deoxycytidine and the histone deacetylase inhibitor tricostatin A. These findings formally indicate that there is a sub-group of the clinically important cancer/testis genes that are unlikely to be activated in clinical therapeutic approaches using hypomethylating agents and it indicates a unique transcriptional silencing mechanism for germline genes in non-germline cells that might provide a target mechanism for new clinical therapies.

## INTRODUCTION

Whilst oncogenesis is driven by a multitude of complex, non-programmed molecular events, there are a number of key features of this process, not least of which is the aberrant activation of genes that would normally be silenced in a given tissue context [1]. The so called cancer/testis (CT) or cancer germline (CG) genes are one such group of genes that are frequently activated in a range of different human cancer types [2-4]. These genes have expression normally restricted to the human germline, many being testis-specific [2-4]. They have come under intense scrutiny since their original identification as the immunological privilege of their normal germline setting means that the proteins they encode can elicit an immunological response when aberrantly produced in cancers and so have exceptional potential in immunotherapeutics [5]; for example, the *NY-ESO-1* gene product has been successfully targeted in an adoptive therapeutic approach to melanoma therapy [6].

Despite this interest, remarkably little is known about the normal germline function of most CT genes. Moreover, it has been demonstrated that germline genes in *Drosophila melanogaster* are required for the oncogenic process and that the human orthologues of these *Drosophila* genes have up-regulated expression in a range of human cancers, although the functional implications for oncogenesis of this up-regulation remains unclear [7,8]. Interestingly, down-regulation of a number of CT genes in human cancer cells results in perturbation of cellular proliferative potential [for example, see 9,10]. These findings open up the exciting possibility that CT genes might encode functions that are required for tumour homeostasis and it has recently been proposed that tumours become ‘addicted’ to these germline factors [11,12], and recently, meiotic factors have been shown to contribute to telomere maintenance in cancer cells via the ALT pathway [13, 14]. The full extent of germline gene requirement is unclear, but these findings expose a new therapeutic opportunity by directly targeting the tumour-associated function of the CT gene products. Additionally, a number of studies have revealed another clinically important feature of CT genes; their expression appears to drive drug resistance as depletion of the gene products results in enhanced sensitization to anti-cancer drugs [for example, see 15] expanding the therapeutic potential of this important class of cancer genes.

Germline gene expression profiling has also recently been demonstrated to have applications in prognostics and patient stratification. In a seminal study, Rousseaux and co-workers demonstrated that expression of a sub-set of germ line genes in some lung cancers delineated patients with aggressive, metastasis prone tumours with poor prognosis [16]; they extended this by indicating that this cohort of patients might benefit from a drug therapeutic regime that had previously been dismissed for more general use in lung cancer patients, indicating that profiling patients for expression of a small sub-set of germline genes could be used in therapeutic decision making. Understanding germline gene expression is also critical as drug-induced augmentation of expression has also been postulated to be a potential enhancer of immunotherapeutics, the rationale being that further up-regulation of a tumour-specific antigen will result in enhanced immunological targeting of the tumour [for example, see 17].

Taking all these factors together reveals the importance of understanding the regulatory mechanisms for somatic germline gene silencing and their aberrant activation in tumours. To date, the regulation of a number of CT genes has been studied and it has been demonstrated that DNA methylation of regulatory elements, such as promoter-associated CpG islands plays a fundamental role in the somatic silencing of these genes and the hypomethylation of these regulatory DNA regions in cancers is linked to gene activation [for example, see 18-23], whereas gene body hypomethylation has been linked to gene down regulation in cancers [24]. Expression of these genes also becomes activated or further up-regulated upon enforced hypomethylation by the DNA methyltransferase inhibitor 5-aza-2′-deoxycytidine (5-aza-CdR), and to date, all CT genes studied have up-regulated expression in response to this chemotherapeutic agent, indicating a commonality in the mechanistic pathway for somatic CT gene silencing [for example, see 18-23].

To date, most of the CT genes whose expression has been studied are located on the X chromosome (X-CT genes) and belong to large paralogous gene families [2-4]. Recently, a computational pipeline combining expressed sequence tag and microarray meta-analyses of the human orthologues of mouse spermatocyte-specific genes revealed a large cohort of new CT genes that were expressed in a broad spectrum of cancer types [25-29]. Unlike the X-CT genes, the majority of these genes are autosomally encoded and are single copy. To date, the clinical potential of these genes remains largely unexplored. In this current study, analysis of the expression of a small sub-set of these genes reveals a novel feature of CT genes, which indicates that some have a unique mechanism for somatic transcriptional silencing. This is a significant finding as these genes and their associated gene products have an increased prominence in clinical applications and hence the sub-classification of CT genes will play an important role in diagnostics, stratification and therapeutics.

## RESULTS

**Figure 1:**
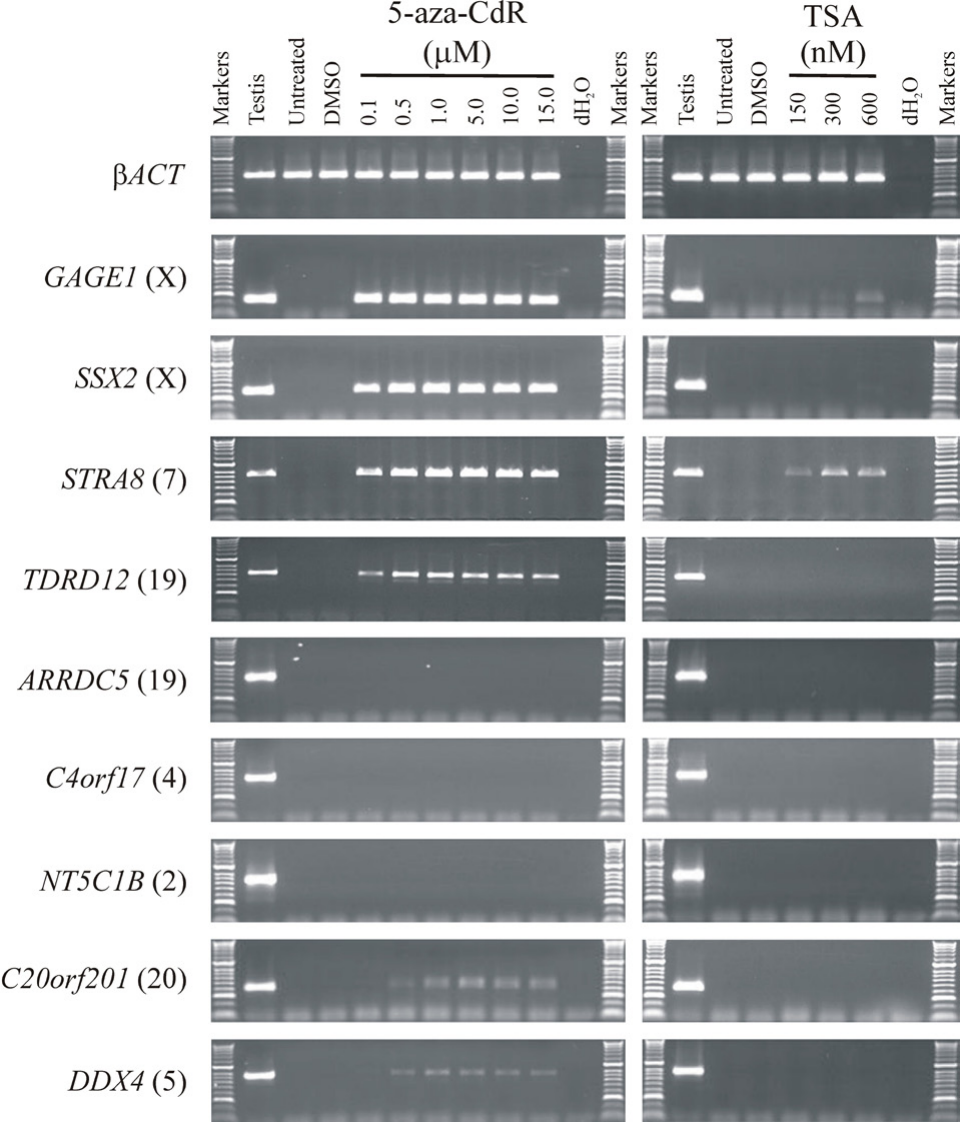
A sub-group of germline genes remain refractory to activation by epigenetic modulating agents RT-PCR was used to analyse activation of a group of germline genes that are normally silenced in the cancer cell line HCT116 (an additional colorectal cell line gives similar results [see Supplementary Figure S1)]. Whilst a cohort of known and newly identified germline genes become activated at low doses of the demethylating agent 5-aza-CdR (*GAGE1, SSX2, STRA8, TDRD12*) and others become activated with slightly higher levels of 5-aza-CdR (*C20orf201, DDX4*), some remain tightly silenced, even at high concentrations of 5-aza-CdR (*ARRDC5, C4orf17, NT5C1B*) (left column). The histone deacetylase inhibitor trichostatin A (TSA) has little activating potential (other than for *GAGE1* and *STRA8*, indicating the primary epigenetic regulation is mediated by DNA methylation (right column). Untreated and DMSO treated cells exhibit no activation of any of the genes analysed for expression activation. The chromosomal location of each gene is provided in parentheses to the right of the gene name. RT-PCR of β*ACT* shows uniform sample quality and loading.

**Figure 2:**
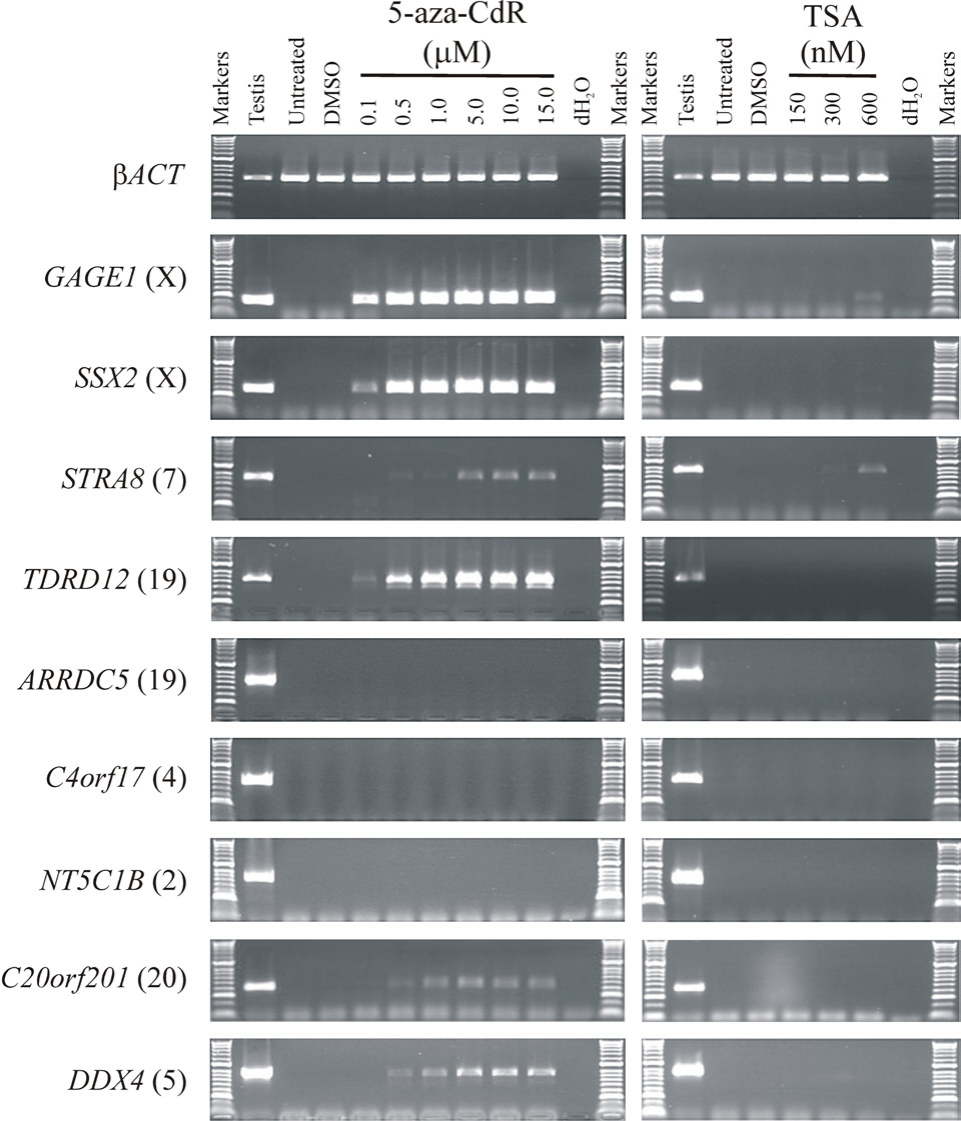
Analysis of germline gene expression in response to epigenetic de-regulation in SW480 human colorectal cancer cells The analysis of expression of a number of germline genes is shown. The agarose gels show RT-PCR products for nine germline genes, including two control germline genes (***GAGE1*** and ***SSX2***) and the β***ACT*** gene as a quality control marker. All germline genes are normally expressed in the testis tissue, but silenced in untreated SW480. The SW480 cells were treated with ranges of concentrations of the epigenetic activators 5-aza-CdR (left hand column) and TSA (right hand column). dH_**2**_O replaced cDNA as a negative control. Gene names are provided to the left of the agarose gel images and the chromosomal location of each germline gene is given in parentheses. The specific concentrations of 5-aza-CdR and TSA are given above the appropriate lane.

**Figure 3:**
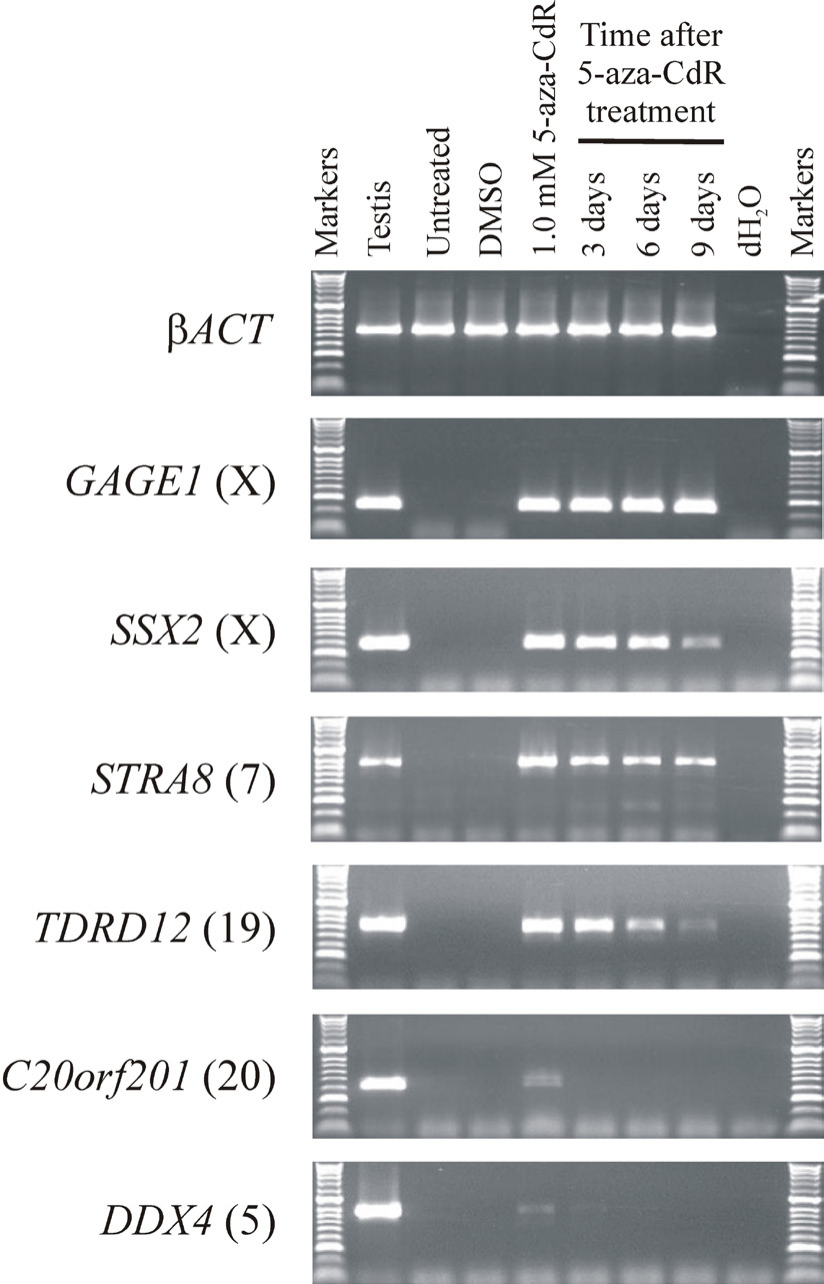
Somatically silenced germline genes that are activated by 5-aza-CdR exhibit differential re-silencing profiles after 5-aza-CdR withdrawal RT-PCR was used to analyse the re-silencing of activated germline genes following removal of the activating agent 5-aza-CdR. Analyses shown are for HCT116 cells. ***GAGE1*** remained highly active following 9 days post 5-aza-CdR removal. RT-PCR indicates ***SSX2, STRA8*** and ***TDRD12*** expression was gradually diminished following the removal of the demethylating agent. Expression of ***C20orf201*** and ***DDX4*** was rapidly lost following removal of 5-aza-CdR. Untreated and DMSO treated cells exhibit no activation of any of the genes analysed for expression activation. The chromosomal location of each gene is provided in parentheses to the right of the gene name. RT-PCR of β***ACT*** shows uniform sample quality and loading.

All CT genes studied to date (mostly X-CT genes) require hypermethylation of regulatory DNA sequences for somatic silencing and are activated by the hypomethylating agent 5-aza-CdR. Given the clinical potential of enhanced up-regulation of immunogenic CT antigens, we set out to explore whether a similar DNA hypermethylation silencing mechanism was operating in the recently identified autosomally encoded CT genes [25,27]. To do this, we selected a small sub-group of these genes that remained transcriptionally silenced in the colorectal cancer cell lines HCT116 and SW480 (*ARRDC5, C4orf17, C20orf201, DDX4, NT5C1B, STRA8, TDRD12*). We also selected two previously characterized CT genes (both X-CT genes) that remained transcriptionally silenced in these two cell lines to serve as exemplar controls for hypermethylation regulated CT genes, *SSX2* and *GAGE1*. To determine whether the novel CT genes are silenced via hypermethylation mediated mechanisms, similar to the characterized X-CT genes, we treated the two cell lines with the DNA methyltransferase inhibitor 5-aza-CdR to determine whether inhibition of DNA methyltransferase activity can activate these genes. Following 5-aza-CdR treatment of HCT116 and SW480 we made cDNA and carried out RT-PCR and agarose gel electrophoresis analysis of the products. The two X-CT genes were activated from the silent state with relatively low levels of 5-aza-CdR (0.1 μM; Figure 1; Figure 2). Some of the novel, autosomally encoded CT genes were similarly activated (*C20orf201, DDX4, STRA8, TDRD12*), although *C20orf201* and *DDX4* required a slightly higher 5-aza-CdR concentration for activation (0.5 μM; Figure 1; Figure 2). Additionally, activation of *STRA8* requires slightly higher concentrations of 5-aza-CdR in SW480 (Figure 2) than HCT116 (Figure 1), which indicates subtle regulatory differences between tumour cell types. However, surprisingly, three genes (*ARRDC5, C4orf17, NT5C1B*) remained tightly transcriptionally silenced, even at high concentrations of 5-aza-CdR in both cell lines (15.0 μM; Figure 1; Figure 2). This unexpected result reveals an important distinction in the way CT gene silencing is epigenetically regulated, revealing a hypermethylation-independent pathway. Interestingly, the X-CT genes (*GAGE1, SSX2*) remained activated for a prolonged period following removal of the hypomethylating agent, as did the autosomally encoded CT genes that were activated with the lowest concentration of 5-aza-CdR (*STRA8, TDRD12*) (Figure 3); however, the other two autosomally encoded CT genes, *C20orf201* and *DDX4*, which required slightly higher concentrations of 5-aza-CdR for activation, reverted to the silent state relatively soon after removal of the hypomethylating agent (Figure 2). This indicates a much greater transcriptional elasticity to the methylation-dependent silencing mechanisms for some CT genes.

To determine whether the silencing of hypermethylation-independent genes (*ARRDC5, C4orf17, NT5C1B*) was mediated via histone deacetylation we also treated the HCT116 and SW480 cells with the histone deacetylase (HDAC) inhibitor trichostatin A (TSA) (Figure 1; Figure 2) or a combination of 5-aza-CdR and TSA (data not shown). Remarkably, all three genes (*ARRDC5, C4orf17, NT5C1B*) remained tightly silenced under these highly transcriptionally permissive conditions.

## DISCUSSION

CT antigens are potentially powerful targets for therapeutics, including immunotherapeutics. However, intratumour CT antigen gene expression is often heterogeneous and so there will be a lack of uniformity for any targeting strategy. To overcome this, it has been demonstrated that treatment of tumours with agents that deregulate epigenetic silencing, such as agents that result in DNA hypomethylation can generate a uniform expression of CT antigen genes within a tumour [for example, see 18-23]. However, to date, the epigenetic regulation mechanisms for CT gene silencing has been restricted to a limited number of X-CT genes, all of which are activated by hypomethylating agents. Here we extended the analysis of epigenetic regulation of clinically important biomarkers and reveal that there is a cohort of CT genes that is not activated in response to hypomethylating agents (or HDAC inhibitors). This regulation is not simply due to a lack of methylation target CpG islands within the promoter regions as at least two of the genes (*ARRDC5* and *NT5C1B*) have reported CpG islands in their transcriptional promoter regions [https://genome.ucsc.edu/]. These observations indicate that there is a very broad range of mechanisms controlling CT gene regulation. This has implications for CT gene selection for clinical targeting strategies. Moreover, the mechanistic regulatory pathways might indicate sub-groups of CT genes that are co-regulated, which has implications for the study of these genes both as biomarkers, potential oncogenes and/or encoders of drug targets. Additionally, it has been demonstrated that some CT genes are required for tumour cell proliferation. Turning off these genes could reduce the proliferation-mediated burden of tumours, restricting their disease effect and/or enhancing other therapeutic approaches.

## MATERIALS AND METHODS

### Maintenance and culturing of human colorectal cell lines HCT116 and SW480

HCT116 and SW480 cell lines were obtained from the European Collection of Cell cultures. Both lines are tested for authenticity once per annum by LGC StandardsTM (authentication tracking number 710236782). HCT116 cells were grown in McCoy's 5A medium with GLUTAMAX^TM^ (Invitrogen, GIBCO 36600) and SW480 cells were grown in Dulbecco's modified Eagle's medium with GLUTAMAX^TM^ (Invitrogen, GIBCO 61965). Both media types were supplemented with 10% foetal bovine serum (Invitrogen; GIBCO 10270). Cells were incubated in humidified incubators at 37°C in a 5% CO_2_ atmosphere.

Cells cultures were tested for mycoplasma infection using the LookOut^TM^ Mcycoplasma PCR Detection kit (Sigma Aldrich, MP0035). Epigentics modulating agents were added to the concentrations required as indicated in the main text. Treatment with 5-aza-CrD and TSA was for 48 hours (72 hour treatment yielded identical results).

### RNA extraction, cDNA synthesis and polymerase chain reaction

Total RNA was isolated using Trizol reagent (Invitrogen; 15596-026). Confluent cells were homogenised in Trizol (1 ml Trizol / 5×10^6^ cells) and held at room temperature (RT) for 5 minutes. Chloroform (200 μl per 1 ml of Trizol) was added to each sample and the homogenate was vigorously shaken for 15 seconds, followed by incubation for 5 minutes at RT. Samples were then centrifuged at 12,000 *g* for 15 minutes at 4°C. The aqueous layer was then removed to a new Eppendorf tube and 500 μl of isopropanol was added. After incubation at RT for 10 minutes, the samples were centrifuged again at 12,000 *g* for 20 minutes. The supernatant was removed and the pellet was washed with 70% ethanol and re-centrifuged at 7,500 *g* for 5 minutes at 4°C. The supernatant was discarded again and the cell pellet was left to dry at RT for 5-10 minutes, and then 100 μl RNase free water containing 2 μl DNase I (Sigma; D5319) was added to each RNA preparation sample. The samples were incubated at 37°C for 10 minutes and then at 75°C for 10 minutes. RNA quality and concentration was measured with a NanoDrop (ND 1000) spectrophotometer.

### Total RNA from normal human testis tissues was supplied by Clontech (Catalogue number; 636643)

Total RNA was used to synthesise cDNA using a SuperScript III First Strand Synthesis Kit (Invitrogen; 18080-051). Samples (1-2 μg) of total RNA were used according to the manufacturer's protocol. PCR using β*ACT* primers was used to check the cDNA quality.

Gene sequences were obtained from the National Center for Biotechnology Information (http://www.ncbi.nlm.nih.gov). Primers were designed to span more than one intron where possible. Primers were designed using Primer 3 software (http://primer3.ut.ee/). Primer sequences are provided in the Supplementary Materials.

For PCR amplification, 2 μl of diluted cDNA was supplemented with 25 μl of BioMixTM Red (Bioline; BIO-25006) and 1 μl each of the forward and reverse primer, and the final volume was adjusted with ddH_2_O to 50 μl. PCR for samples was initiated with a pre-cycling melting step at 96°C for 5 minutes, followed by 40 cycles of denaturing at 96°C for 30 seconds, an annealing step was carried out between 58-62°C for 30 seconds (specific annealing temperatures are provided in the table below), extension at 72°C for 30 seconds and the final extension temperature was 72°C for 5 minutes. All PCR products were evaluated on 1% agarose gels stained with ethidium bromide

**Table:** PCR Primer sequence

Gene	Forward primer sequence (5′-3′)	Reverse primer sequence (5′-3′)	PCR annealing Temp. (°C)
*βACT*	TGCTATCCCTGTACGCCTCT	CGTCATACTCCTGCTTGCTG	58.0
*GAGE1*	TAGACCAAGGCGCTATGTAC	CATCAGGACCATCTTCACAC	58.4
*SSX2*	CAGAGATCCAAAAGGCC	CTCGTGAATCTTCTCAGAGG	58.4
*ARRDC5*	CAACAAGGCAGACTACGTGC	GCGAGTGTGCATGATCTCAC	60.5
*C4orf17*	CCTCATCCCAGAAGAGTCTG	CTGCTGCTGGTTCCATTGAG	60.5
*C20orf201*	ATCTGCTCTTCGGCGACCTG	ACACTCTCAGTCGCCGTCAC	60.0
*DDX4*	GTGCTACTCCTGGAAGACTG	CCAACCATGCAGGAACATCC	60.5
*NT5C1B*	CGGCAGGAAAATCTACGAGC	CTGTAACCAGGTAGGTCCTG	60.5
*STRA8*	TGGCAGGTTCTGAATAAGGC	GAAGCTTGCCACATCAAAGG	58.4
*TDRD12*	GAGCTAAAGTGCTGGTGCAG	CTGAGGTCACCGACAATACC	60.5

## References

[1] Wang J, Rousseaux S, Khochbin S (2014). Sustaining cancer through addictive ectopic gene activation. Curr Opin Oncol.

[2] Fratta E, Coral S, Parisi G, Colizzi F, Danielli R, Nicolay HJ, Siqalotti L, Maio M (2011). The biology of cancer testis antigens: putative function, regulation and therapeutic potential. Mol Oncol.

[3] Simpson AJ, Caballero OL, Jungbluth A, Chen YT, Old LJ (2005). Cancer/testis antigens, gametogenesis and cancer. Nat Rev Cancer.

[4] Whitehurst AW (2014). Causes and consequences of cancer/testis antigen activation in cancer. Annu Rev Pharmacol Toxicol.

[5] Mellman I, Coukos G, Dranoff G (2011). Cancer immunotherapy comes of age. Nature.

[6] Hunder NN, Wallen H, Cao J, Hendricks DW, Reilly JZ, Rodmyre R, Jungbluth A, Gnjatic S, Thompson JA, Yee C (2008). Treatment of metastatic melanoma with autologous CD4+ T cells against NY-ESO-1. N Eng J Med.

[7] Janic A, Mendizabal L, Llamazares S, Rossell D, Gonzalez C (2010). Extopic expression of germline genes drives malignant brain tumour growth in Drosophila. Science.

[8] Feichtinger J, Larcombe L, McFarlane RJ (2014). Meta-analysis of expression of l(3)mbt tumour-associated germline genes supports the model that a soma-to-germline transition is a hallmark of human cancer. Int J Cancer.

[9] Linley AJ, Mathieu MG, Miles AK, Rees RC, McArdle SE, Regad T (2012). The helicase HAGE expressed by malignant melanoma-initiating cells is required for tumour cell proliferation *in vivo*. J Biol Chem.

[10] Cappell KM, Sinnott R, Taus P, Maxfield K, Scarbrough M, Whitehurst AW (2012). Multiple cancer testis antigens function to support tumour cell mitotic fidelity. Mol Cell Biol.

[11] Rousseaux S, Wang J, Khochbin S (2013). Cancer hallmarks sustained by ectopic activations of placenta/male germline genes. Cell Cycle.

[12] McFarlane RJ, Feichtinger J, Larcombe L (2014). Cancer germline gene activation: Friend or foe?. Cell Cycle.

[13] Arnoult N, Karlseder J (2014). ALT telomeres borrow from meiosis to get moving. Cell.

[14] Cho NW, Dilley RL, Lampson MA, Greenberg RA (2014). Interchromosomal homology searches drive directional ALT telomere movement and synapsis. Cell.

[15] Whitehurst AW, Bodemann BO, Cardenas J, Ferguson D, Girard L, Peyton M, Minna JD, Michnoff C, Hao W, Roth MG, Xie XJ, White MA (2007). Synthetic lethal screen identification of chemosensitizer loci in cancer cells. Nature.

[16] Rousseaux S, Debernardi A, Jacquiau B, Vitte AL, Vesin A, Nagy-Mignotte H, Moro-Sibilot D, Brichon PY, Lantuejoul S, Hainaut P, Laffaire J, de Reynies A, Beer DG (2013). Ectopic activation of germline and placental genes identifies aggressive metastasis-prone lung cancers. Sci Trans Med.

[17] Karpf AR (2006). A potential role for epigenetic modulatory drugs in the enhancement of cancer/germ-line antigen vaccine efficacy. Epigenetics.

[18] De Smet C, Lurquin C, Lethe B, Martelange V, Boon T (1999). DNA methylation is the primary silencing mechanism for a set of germ line- and tumor-specific genes with a CpG-rich promoter. Mol Cell Biol.

[19] Fratta E, Sigalotti L, Colizzi F, Covre A, Nicolay HJMG, Danielli R, Fonsatti E, Altomontet M, Calabro L, Coral S, Maio M (2010). Epigenetically regulated clonal heritability of CTA expression profiles in human melanoma. J Cell Physiol.

[20] Mossman D, Scott RJ (2011). Long term transcriptional reactivation of epigenetically silenced genes in colorectal cancer cells requires DNA hypomethylation and histone acetylation. PLoS One.

[21] Cannuyer J, Loriot A, Parvizi GK, De Smet C (2013). Epigenetic hierarchy within the MAGEA1 cancer-germline gene: promoter DNA methylation dictates local histone modifications. PLoS One.

[22] James SR, Cedeno CD, Sharma A, Zhang W, Mohler JL, Odunsi K, Wilson EM, Karpf AR (2013). DNA methylation and nucleosome occupancy regulate the cancer germline antigen gene MAGEA11. Epigenetics.

[23] Kim R, Kulkarni P, Hannenhalli S (2013). Derepression on cancer/testis antigens in cancer is associated with distinct patterns of DNA hypomethylation. BMC Cancer.

[24] Yang X, Han H, De Carvalho DD, Lay FD, Jones PA, Liang G (2014). Gene body methylation can alter gene expression and is a therapeutic target in cancer. Cancer Cell.

[25] Feichtinger J, Aldeailej I, Anderson R, Almutairi M, Almatrafi A, Alsiwiehri N, Griffiths K, Stuart N, Wakeman JA, Larcombe L, McFarlane RJ (2012). Meta-analysis of clinical data using human meiotic genes indicates a novel cohort of highly restricted cancer-specific marker genes. Oncotarget.

[26] Feichtinger J, McFarlane RJ, Larcombe LD (2012). CancerMA: a web-based tool for automatic meta-analysis of public cancer microarray data. Database.

[27] Sammut SJ, Feichtinger J, Stuart N, Wakeman JA, Larcombe L, McFarlane RJ (2014). A novel cohort of cancer-testis biomarker genes revealed through meta-analysis of clinical data sets. Oncoscience.

[28] Feichtinger J, McFarlane RJ, Larcombe LD (2014). CancerEST: a web-based tool for automatic meta-analysis of public EST data. Database.

[29] Lafta IJ, Bryant HE, Goldman AS (2014). ‘Sex’ in the cancer cell. Oncotarget.

